# Now More Than Ever: Considering Health System Reforms in the Post-COVID 19 Scenario

**DOI:** 10.1007/s41027-020-00272-z

**Published:** 2020-09-16

**Authors:** Sumit Mazumdar

**Affiliations:** Centre for Health Economics, University of York, York, UK

As the world continues to reel under the unprecedented impacts of the global pandemic triggered by the novel coronavirus (COVID-19) that has hardly left countries, regions and populations with their familiar course of life and living, it also provides useful lessons on how public systems can be made more resilient and responsive. An important aspect of the recent experience is to assess how health systems—particularly in low-resourced settings as in most low- and lower-middle-income countries—have responded to manage the highly dynamic, uncertain challenges posed by the pandemic. As in most other key development sectors for large, diverse countries such as India, it is also of prime importance for health systems to be able to strike a critical balance: adapt to the new paradigm of health care needs following from the pandemic through appropriate measures and mechanisms but also remain cognizant of other priorities and commitments across the health sector. This calls for doing more and also doing better, particularly for appropriate stewardship by the government backed by political will. This essay briefly outlines how some of the key learnings in course of responding to the pandemic in India can be incorporated in adaptive reform measures for the health system that aims to ‘build back better’.^1^

## Evident Fault Lines Across the Health System

Undoubtedly, it is a tough task for any health system to organise, provide, finance and coordinate health services in the Indian context, with its immense regional, socioeconomic, cultural, political and administrative diversities. Researchers and commentators have observed several challenges around India’s contemporary experience of pursuing goals of positive health outcomes, adequate standards of quality of care and ensuring protection against financial risks arising out of ill-health and medical care, in a manner that prevents or reduces risks of inequity or health-induced poverty traps. For a country of its India’s size, population density and generally poor public health standards, containing the spread and fatality of an unknown pandemic as COVID-19 has been always a formidable challenge. Although questions have been raised regarding the actual scale of the pandemic and consequent mortality that might be masked due to low levels of testing and not having comprehensive systems of surveillance across the country, it is apparent that immediate casualties have been kept under manageable levels by the health system and wider supportive actions such as the ‘lockdowns’ and other restrictions on normal economic and social activities. However, it has massively stretched the system revealing several fault lines in how the health system responds to an emergency, simultaneously not affecting routine but critically important actions.

Health systems are generally considered to be an interrelated connection between six ‘building-blocks’—service delivery, medical equipment, manpower or health workforce, information, financing and stewardship or coordinated leadership. In India, as in many other similar contexts, the pandemic has exposed long-standing weaknesses across these domains, and only intensifying in face of the emergency. The pandemic has revealed the problems a system has to deal with if referral mechanisms through appropriate cascade of care involving stages of screening, testing, preventive quarantine, hospitalisation and critical care are not synchronised; while some regions and metropolitan cities with better wherewithal have been able to have some amount of coordinated patient flows, elsewhere this has led to much chaos and confusion both among patients and providers. An almost entirely market-driven and market-led system that controls supplies of almost all critical inputs for medical care—drugs and other medical supplies including diagnostic kits, protective equipment and even patient transportation—and poor regulatory oversight by governments has resulted in frequent instances of shortages in supply, skewed distribution of essential inputs and resultant spikes in their prices. Apart from major urban centres, adequate infrastructure of physical and human resource has also been found wanting; most facilities of critical care—specialist physicians and paramedics, life support equipment—being almost exclusively concentrated in state capitals or metro cities have only resulted in an overstretched health workforce and costly delays in treatment. In most states, except for the few private hospitals requisitioned by governments for treating patients with COVID-19, there has been little coordination between the public and the private sector, mostly resulting in several reported instances of malpractices by the later such as refusal of treatment or charging exorbitant prices. Financing of health care, in classic textbook illustrations of market failures, has continued to be dominated by regressive out-of-pocket payments even under coverage of private and public health insurance programmes such as the Pradhan Mantri Jana Aarogya Yojana (PMJAY). In a curious case of adverse selection, several private hospitals have been allegedly reported to turn away patients having health insurance coverage on different pretexts, but mostly to escape scrutiny of hospitals bills by insurance companies or administrators. Throughout the unfolding of the pandemic and its different stages, there have been several instances of improper coordination between different government agencies in reporting the cases and casualties, little-helped by contradictory statements, at times with weak or no scientific evidence. Finally, while the pandemic has vindicated how strong, decentralised governance can be effective in responding to such emergencies in a timely and coordinated manner, there has been some tendency to centralise several key aspects of decision-making and setting priorities at local levels.

## Lessons Learnt from the Pandemic and the Health System’s Response

Although, the pandemic is far from being past its prime amidst all uncertainties regarding its prognosis as well as effectiveness and sustainability of the countering responses, a few important lessons for the health system are already evident.

Firstly, *key inputs to the health system face infrastructural constraints, weak distribution mechanisms and are highly fragmented across roles and between actors*. These call for a pressing priority of harmonising how these different inputs are produced and distributed across the system. In a market-oriented health system, poorly defined or inadequate supply-side incentives in the sub-markets for these inputs—health workforce; medicines, vaccines and medical equipment; physical infrastructure—lead to skewed supply of these inputs and increase inequity in access to, use of and financing health care. Regulatory mechanisms in key input sectors, e.g. medical and nursing colleges, pharmaceuticals and medical equipment, clinical establishment in general, that remain ambiguously defined, weakly enforced and suffer from duplication of functions and lack of transparency are major barriers to ensure efficient functioning of the different markets for health care. Among the different inputs of similar nature, some has been prioritised over other without considering both demand and supply constraints—health workforce being a case in point. Excessive preoccupation with medical colleges and training specialist physicians has led to a crippling neglect of training paramedical workers and other medical technicians, notwithstanding their critical importance in emergency response. More than 70 years of coexistence of the public and private sectors in most of these input markets have defied both social as well as economic logic with little improvement in coordination among roles and functional mechanisms.

Secondly, a *highly* *fragmented system of financing health care leads to both inefficiency for the system and inequity for the people* which only accentuates in emergencies. The flagship national health insurance programme—the Ayushman Bharat—Pradhan Mantri Jana Aarogya Yojana (AB-PMJAY)—launched in 2018 with a ‘target’ of covering 100 million families for cashless hospitalisation and currently covering about a tenth of that figure, is yet to make its impact. Although, still in its early days of implementation, studies^2^ suggest limited effectiveness of AB-PMJAY to extend financial risk protection, with reports indicating only a handful availing its benefits for COVID-19.^3^ The AB-PMJAY coexists with several other private health insurance schemes available to those who can afford to pay the premiums and employer-provided schemes such as for government employees or formal sector workers, but in effect, leaves out vast numbers—including the poor as well as millions of ‘middle-class’ families missing the coverage criteria of government schemes and unable to afford private insurance—without any effective means to insure against adverse financial implications associated with medical care. Although recent efforts are being undertaken under AB-PMJAY to standardise payment mechanisms across the country, inefficiencies across the system and inequity in financing is unlikely to reduce if different groups of people continue to be covered differently, and some not at all.

Thirdly, *effectiveness of policies and interventions are critically dependent on local capacities, coordination and flexibility* of decision-making across different public agencies and with the private sector on the one hand and between the different levels of public administration within health department, on the other. This includes transparent and accountable systems of collecting and disseminating information, including key health statistics, and ability for priority-setting at local levels. Across the world, responding to the COVID-19 challenges has been the most successful in decentralised health systems working synchronously with decentralised governance systems at large. In India, the National Rural Health Mission (NRHM) (and its successor National Health Mission (NHM) had aimed at strengthening decentralised functioning of the health system with institutionalised links with local political leadership, but seem to have lost its steam after more than a decade in operation, without ensuring the goals achieved or the lessons learnt. The way forward is not of abandoning the rudimentary decentralised system that has been emerging out of the NRHM/NHM but strengthening it through sustained investment and support in technical, management and functional capacities of both local level health and political functionaries.

## Key Considerations for Progressive and Adaptive Health System Reforms

Across the world, the COVID-19 pandemic has provoked public policy to be reconsidered in a different paradigm of how public action needs to be dynamically adapted to both persisting questions as well as new, unprecedented threats to the development processes. In countries such as India, while the crisis has revealed deep fault lines within the health system and its linkages with other development sectors, it also provides an opportunity to calibrate much-needed people-centric and adaptive reforms across the health system. Drawing parallels with post-disaster reconstruction involving different actions and actors, this calls for ‘building back better’.

First, *capacity of health systems*—in terms of key physical, manpower and financial resources—that allows responding to emergencies without destabilising other concurrent priorities *needs to be significantly strengthened*. Uninterrupted actions in providing essential services for programmes on controlling infectious diseases such as tuberculosis, malaria, dengue and HIV/AIDS, antenatal and safe delivery care for pregnant mothers, childhood immunization and nutrition, diagnosis and treatment for major non-communicable diseases are critical to ensure sustained progress and avoid costly unmet need. Appropriate and realistic systems of combining incentives and regulatory actions are critical to correct supply-side distortions for most health system inputs and skewed concentration in its distribution.

Second, *reducing fragmentation within and across the key health system building blocks*—service delivery, manpower, financing, availability of medical equipment and drugs—needs to be a foremost priority. An important consideration here is adequate consideration about the ‘missing-middle’ on both supply and demand-side of the system; strengthening infrastructural capacities in districts in a way that most medical care needs can be addressed without the need for travelling out of the district and accounting for financial risk protection for the millions outside of any formal insurance mechanisms that avoids regressive out-of-pocket financing of medical care. Integrating different schemes that moves towards a single system for all Indians regardless of their employment or residential status needs to move beyond rhetoric in a systematic, time-bound manner.

Third, *investing in a transparent, autonomous and robust health information architecture* lies at the core of an adaptive health system. Existing systems such as the Integrated Disease Surveillance Programme (IDSP) and the Civil Registration System need radical reforms to improve coverage, reporting and data quality. Encouraging new mechanisms such as electronic health records and harmonising existing reporting systems across health facilities—including the organised private sector—that leverages from a strong Information Technology (IT) backbone and data protection regulations remains a key feature of any health reform strategy.

Finally, the pandemic has brought to the fore several *new questions and considerations for the health system* to respond. This includes issues such as enforcing physical distancing, personal hygiene and other essential protocols in health facilities under high patient loads; ensure equitable, transparent systems of access to COVID-19 medicines and vaccine(s); specialised needs for other pandemic-induced conditions, particularly mental health care needs, including among children and adolescents and accounting for intensified vulnerability due to adverse economic implications of the pandemic. This requires effective stewardship roles by the government to coordinate actions between the public and the private sectors particularly in service delivery and medical supplies, and also between public health and other different non-health government departments.

None of these are possible without transformative increases in public investments. Notwithstanding repeated ‘commitments’ to increase public spending on health, India has been unable to garner the required political will to put more money into health and develop a supportive ecosystem that ensures it is spent well. Health policy is inherently political as it decides who gets what and in what manner. Now more than ever, it is critical that political will in India assumes an assertive and rightful way in steering the health system on the road to responsive reforms.

## Footnotes

1 Drawing from the post-disaster recovery literature, Building Back Better (BBB) approaches advocate reconstructing health and other systems in more responsive and efficient ways. A recent paper discusses how BBB approaches need to be integrated with universal health coverage, see https://www.uhc2030.org/fileadmin/uploads/uhc2030/Documents/Key_Issues/Health_emergencies_and_UHC/UHC2030_discussion_paper_on_health_emergencies_and_UHC_-_May_2020.pdf.

2 For, e.g. see Garg et al (2020) available at https://bmcpublichealth.biomedcentral.com/track/pdf/10.1186/s12889-020-09107-4.

3 https://www.thehindu.com/news/national/only-2132-availed-or-being-treated-for-COVID-19-under-ab-pmjay/article31635083.ece

